# Systolic blood pressure values might further risk-stratify the adverse outcomes of LVH in older patients with chronic kidney disease

**DOI:** 10.1186/s40885-016-0056-7

**Published:** 2016-11-23

**Authors:** Carlos R. Franco Palacios, Pankaj Goyal, Amanda M. Thompson, Brent Deschaine

**Affiliations:** 1 Nephrology Department, Affiliated Community Medical Centers, Rice Memorial Hospital, 101 Willmar 274 Ave SW., Willmar, MN 56201 USA; 2 Hospital Medicine Department, Affiliated Community Medical Centers. Rice Memorial Hospital, Willmar, MN USA; 3 Pharmacy Department, Rice Memorial Hospital, Willmar, MN USA; 4 University of Oklahoma, Cardiac Catheterization Laboratory, Oklahoma, USA

**Keywords:** Hypertension, Chronic kidney disease, Elderly, Cardiovascular disease, Outcomes, LVH

## Abstract

**Background:**

LVH is highly prevalent in patients with CKD and is independently associated with subsequent cardiovascular events.

We hypothesized that adding systolic blood pressure values to LVH might differentiate different subgroups of patients at higher risk of cardiovascular events (CVE) and other adverse outcomes.

**Methods:**

Retrospective cohort study of 243 patients older than 60 years with stages 1-5 pre-dialysis CKD. LVH was assessed by electrocardiogram or echocardiogram.

**Results:**

Cardiovascular events occurred in 7 patients (10.3%) among those with SBP <130 and no LVH, 8 patients (10.5%) among those with SBP ≥130 and no LVH, 7 patients (21.2%) among those with SBP <130 and LVH and 25 patients (37.9%) among those with SBP ≥ 130 and LVH.

On multivariate analyses, comparing to SBP < 130 and no LVH, the HR for CVE in those with SBP ≥ 130 and LVH was 4 (1.75, 10.3), *p* = 0.0007; 2.13 (0.71, 6.32) *p* = 0.16 in those with SBP <130 and LVH and 1.20 (0.42, 3.51) *p* = 0.72 in those with SBP ≥130 and no LVH.

No significant differences were noted in changes in renal function and mortality rates among the groups.

**Conclusion:**

The combination of higher systolic blood pressure and LVH might identify older patients with CKD at higher risk of cardiovascular outcomes.

## Background

Cardiovascular disease represents the main cause of morbidity and mortality in patients with chronic kidney disease (CKD).

Left ventricular hypertrophy (LVH) is a known manifestation of hypertensive target organ damage.

LVH is highly prevalent in patients with CKD and is already present in the early stages of the disease. Its prevalence increases with age, anemia, hypertension and lower renal function [1, 2].

More advanced CKD at baseline may be associated with larger longitudinal increases in left ventricular mass and volume and greater deterioration in diastolic function [3].

On the other hand, more severe LVH is associated with kidney function decline and progression to dialysis [4, 5].

In patients with CKD, LVH and other echocardiographic parameters (including low EF) are independently associated with subsequent cardiovascular events [6, 7].

Because of the above, treating hypertension is of paramount importance in CKD populations. At the same time, the ideal blood pressure target in these patients is a subject of controversy. Retrospective studies in older patients with CKD suggest mortality is increased for systolic blood pressures (SBP) higher and lower than 130-139 mmHg and DBP of 60-79 mmHg [8].

The objective of this study was to assess the prognostic value of combining LVH data with clinical data (SBP) as markers of adverse outcomes in older patients with CKD.

Our hypothesis is that adding systolic blood pressure values to LVH might help differentiate different subgroups of patients at higher risk of adverse outcomes.

## Methods

After obtaining approval from the Rice Memorial Hospital Institutional Review Board (Willmar MN), a retrospective cohort study of 243 patients older than 60 years with stages 1-5 pre-dialysis CKD was carried out. These patients were seen in a Nephrology clinic between January of 2013 and November of 2015.

Since prior studies in older patients with CKD suggest that mortality is lower for a SBP around 130 mmHg, we decided to use this value as reference [8].

*CKD* was defined as evidence of structural or functional kidney abnormalities (abnormal urinalysis, imaging studies, or histology) that persisted for at least 3 months, with or without a decreased glomerular filtration rate (GFR), as defined by a GFR of less than 60 mL/min per 1.73 m2.

### Exclusion criteria

Patients on renal replacement therapy, history of organ transplant, patients younger than 60 years of age, patients with a clinical diagnosis of congestive heart failure (CHF) at baseline, patients without LVH data.

### Variables collected

Demographics, comorbidities, LVH assessment by electrocardiogram (ECG) or echocardiogram and laboratory data at baseline and during follow up.

### Primary outcome: new cardiovascular events

*Cardiovascular events (CVE*) were defined as the composite of new episodes of acute coronary event, decompensated heart failure and cerebrovascular accident (stroke, transient ischemic attack) during the follow up period.

### Secondary outcomes

End stage renal disease requiring dialysis; changes in GFR; all-cause mortality. The cause of death was obtained from the medical records or death certificates.

### Estimation of blood pressure

Office systolic blood pressures (SBP) and diastolic blood pressures (DBP) were measured with an automated device, using an adult cuff, with the patient in a seated position. Pulse pressure was determined by subtracting the DBP from the SBP. In the calculation of the mean blood pressure, we used blood pressures collected during regular visits to maintain consistency among all participants. A median of 18 [10, 28] blood pressure readings were obtained for each patient.

### Definition of LVH

The patients included in the study had LVH evaluation either by echocardiogram or electrocardiogram (ECG), these reports were obtained from the medical chart. A patient was deemed to have LVH if there was a report (either by ECG or echocardiogram) diagnosing this condition. LVH by echocardiogram is defined as a LVMI (left ventricular mass index) ≥150 g/m^2^ [9].

LVH by electrocardiogram is defined by a Sokolow-Lyon voltage amplitude of (SV1 + RV5 or RV6) ≥3.5 mV and/or a Cornell voltage of (SV3 + RaVL) >2.8 mV.

### Glomerular filtration rate (GFR)

GFR was estimated by the four-variable Modification of Diet in Renal Disease formula (MDRD) [10].

### Estimation of proteinuria

Random urine albumin (albumin-to-creatinine ratio, expressed as mg/g) or total protein (protein-to-creatinine ratio, expressed as mg/g) were obtained after reviewing the medical chart.

### Statistical analyses

Data are presented as mean and standard deviation if normally distributed and median [25% and 75% percentiles] or range if not. For parametric data, differences in the mean were compared by ANOVA. For highly skewed data, the Wilcoxon- Kruskal Wallis Test was used.

Differences in proportions were assessed by the Chi square or Fisher’s exact test.

Survival curves were generated using the Kaplan-Meier method. Cox proportional hazards models were used to study associations and adjust for confounding factors.

*P* values lower ≤0.05 were considered statistically significant. All the analyses were performed using JMP statistical software version 11.2.0 (SAS Campus Drive, Cary, NC).

## Results

The baseline characteristics are depicted in Table 1.

**Table 1:** Baseline characteristics

	SBP < 130 and LVH *N* = 33	SBP ≥130 and LVH *N* = 66	SBP < 130 and no LVH *N* = 68	SBP ≥130 and no LVH *N* = 76	*P* value
Age, ﻿years, mean ± SD	76 ± 7.2	77.4 ± 8.9	72.8 ± 8.2	72.9 ± 8.2	0.002
Caucasian, %	31 (93.9)	65 (98.5)	67 (98.5)	71 (93.4)	0.38
Men, %	15 (45.4)	33 (50)	30 (44.1)	36 (47.4)	0.92
Obesity, %	23 (69.7)	31 (47)	38 (55.8)	38 (50)	0.15
Cancer, %	7 (21.2)	24 (36.4)	13 (19.2)	14 (18.4)	0.05
Ejection fraction, mean ± SD*	61 ± 7.2	60 ± 7.7	58 ± 7.8	61 ± 8.8	0.35
CAD, %	12 (36.3)	22 (33.3)	21 (30.9)	11 (14.5)	0.02
CVA, %	1 [3]	7 (10.6)	4 (5.9)	5 (6.6)	0.51
DM, %	18 (54.5)	32 (48.5)	32 (47)	38 (50)	0.91
HTN, %	29 (87.9)	62 (93.9)	64 (94.1)	71 (93.4)	0.71
A fib, %	8 (24.2)	11 (16.7)	9 (13.2)	10 (13.1)	0.49
Hyperlipidemia, %	24 (72.7)	41 (62.1)	55 (80.9)	56 (73.7)	0.11
Pulmonary disease, %	10 (30.3)	16 (24.2)	13 (19.1)	22 (28.9)	0.49
Chronic liver disease, %	1 [3]	4 [6]	5 (7.3)	2 (2.6)	0.52
ACEIs, %	17 (51.5)	32 (48.5)	29 (42.6)	39 (51.3)	0.73
ARBs, %	4 (12.1)	16 (24.2)	9 (13.2)	15 (19.7)	0.29
Calcium channel blockers, %	7 (21.2)	29 (43.9)	21 (30.9)	35 (46)	0.03
Beta blockers, %	21 (63.6)	46 (69.7)	38 (55.9)	35 (46)	0.03
Thiazide diuretics, %	13 (39.4)	29 (43.9)	29 (42.6)	34 (44.7)	0.96
Loop diuretics, %	11 (33.3)	21 (31.8)	19 (27.9)	24 (31.6)	0.93
K sparing diuretics, %	2 [6]	1 (1.52)	9 (13.2)	3 (3.95)	0.03
Clonidine, %	0 (0)	4 [6]	1 (1.5)	3 (3.95)	0.32
Vasodilators, %	2 [6]	17 (25.8)	7 (10.3)	5 (6.6)	0.004
SBP mmHg, mean ± SD	123.9 ± 4.13	140.5 ± 8	122 ± 5.3	139.3 ± 8.3	<0.0001
DBP mmHg, mean ± SD	69.7 ± 5.52	73.1 ± 7.32	70.9 ± 5.4	74.7 ± 6.6	0.0002
PTH pg/mL, median [IQR]	59 [35, 83]	56 [42, 82]	67 [36, 92]	65 [45, 96]	0.81
Hemoglobin g/dL, mean ± SD	13 ± 1.44	12.4 ± 1.89	12.7 ± 1.66	13 ± 1.84	0.13
Phosphorus mg/dL, mean ± SD	3.35 ± 0.43	4 ± 3.28	3.46 ± 0.66	3.43 ± 0.64	0.31
Bicarbonate mEq/L, mean ± SD	27.9 ± 2.96	27.2 ± 2.94	26.4 ± 3.32	26.9 ± 3.12	0.13
25-Vitamin D ng/mL, median [IQR]	31.8 [23, 43]	36.9 [26, 48.7]	38.7 [21.3, 48.7]	28.9 [19.8, 48.8]	0.54
Albumin g/dL, mean ± SD	4 ± 0.30	4 ± 0.31	4 ± 0.36	4.1 ± 0.32	0.36
Baseline GFR, mean ± SD	48.2 ± 18.1	45 ± 18.8	47.6 ± 2.3	53.3 ± 2.1	0.06
Proteinuria, mg/g, median [IQR]	100 [7, 214]	100 [46, 303]	74.4 [8.4, 104]	100 [16.7, 333]	0.01

*IQR* interquartile range, *GFR* glomerular filtration rate (cc/min/BSA), *SBP* systolic blood pressure, *DBP* diastolic blood pressure

*EF data was obtained in 146 patients

Echocardiographic information was present in 160 patients. Electrocardiographic information was present in 84 patients. Whenever the two were present, the echocardiographic data was used for analyses.

**Fig. 1:**
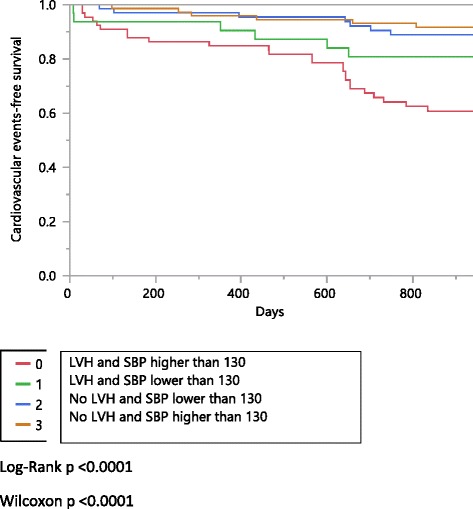
Kaplan-Meier analyses of cardiovascular event-free survival according to LVH and SBP

Primary and secondary outcomes are reported in Table 2. During the follow up period (median 2.5 years, range 0.02-2.8) there were 47 cardiovascular events and 13 deaths. The incidence of cardiovascular events was higher in those patients with LVH, especially in those with a SBP ≥130 mmHg (Fig. 1).

**Table 2:** Primary and secondary outcomes

	SBP < 130 and LVH	SBP ≥130 and LVH	SBP < 130 and no LVH	SBP ≥130 and no LVH	*P* value
CVE, %	7 (21.2)	25 (37.9)	7 (10.3)	8 (10.5)	<0.0001
Death, %	3 (9)	5 (7.58)	4 (5.9)	1 (1.32)	0.25
Changes in GFR during follow up (cc/min/BSA)	-5.27 ± 9.48	-3.57 ± 12.1	-5 ± 15.4	-7.7 ± 13.2	0.31
ESRD, %	1 (3)	4 (6)	0	2 (2.63)	0.22

*CVE* cardiovascular events, *BSA* body surface area, *ESRD* end stage renal disease, *GFR* glomerular filtration rate

Comparing to SBP < 130 and no LVH the unadjusted HRs for CVE in those with SBP ≥ 130 and LVH was 4 (1.82, 10), *p* = 0.0003; 2.18 (0.74, 6.39), *p* = 0.14 in those with SBP < 130 and LVH and 0.96 (0.34, 2.74) *p* = 0.94 in those with SBP ≥ 130 and no LVH.

On multivariate analyses, after adjusting for confounding factors, the association of LVH and SBP ≥130 with CVE remained significant (Table 3).

**Table 3:** Multivariate model for cardiovascular events

	HR (95%CI)	*P* value
SBP <130 and LVH*	2.13 (0.71, 6.32)	0.16
SBP ≥130 and LVH*	4 (1.75, 10.3)	0.0007
SBP ≥130 and no LVH*	1.20 (0.42, 3.51)	0.72

*Using SBP < 130 and no LVH as comparison. Cox models were adjusted for age, CAD, CVA, beta blocker use, vasodilator use, atrial fibrillation, obesity

No significant differences were noted in changes in renal function and mortality rates among the groups, although the number of events was fairly small to make conclusions.

## Discussion

In this study, we found the combination of achieved systolic blood pressure with the presence of LVH (either by ECG or Echocardiogram) might stratify the risk of CVE among older patients with CKD. Furthermore, there appears to be a graded risk, with worse outcomes in those with a SBP ≥130 and LVH vs those with a lower SBP and those without LVH.

Higher blood pressure, greater body mass index (BMI), intravascular expansion, advancing age and male gender are associated with an abnormal left ventricular geometry. This cardiac hypertrophy ultimately leads to activation of metabolic pathways that increase extracellular matrix production leading to fibrosis. Fibrosis leads to reduced contractility with myocardial wall stiffening, systolic and diastolic dysfunction, cardiomyopathy, and congestive heart failure. Changes in cardiac remodeling might be fluid, with some patients reversing to normal geometry, especially with optimal blood pressure control [11–14].

Other principles in the treatment of LVH in CKD patients include treatment of the anemia and secondary hyperparathyroidism.

Unfortunately we did not have enough data to study LV geometry and other echocardiographic parameters in this study. Other studies have found that CV event-free survival is significantly worse in the presence of concentric LVH and eccentric LVH [15].

There is some evidence that a concentric pattern (vs eccentric) portends a worse cardiovascular prognosis. Others suggest that LVH by itself, regardless of the geometry is associated with worse renal and cardiovascular outcomes [16, 17].

The association of hypertension and adverse outcomes has been well demonstrated. In this study we found no difference in survival among the groups, although the number of events is fairly small and definite conclusions cannot be reached.

In the SPRINT trial, aiming for a SBP lower than 120 mm Hg, as compared with less than 140 mm Hg was associated with lower rates of heart failure events, cardiovascular deaths and total deaths [18].

On the other hand, retrospective studies have shown increased mortality with lower blood pressures in the elderly. Kovesdy et al found that patients with a SBP of 130 to 159 mm Hg combined with a DBP of 70 to 89 mm Hg had the lowest adjusted mortality rates, comparing to those with higher or lower BP readings [19].

In 21,000 patients older than 65 years with stage 3-5 CKD not yet on dialysis there was increased mortality with a baseline SBP < 120 mmHg comparing to a SBP between 131-140 mmHg. A SBP higher > 140 mmHg was associated with increased mortality only in the younger patients with CKD [20].

We believe the discrepancy about optimal blood pressure targets in older patients might be related to the presence of CHF, since hypotension in this setting has been associated with reduced survival [21–24].

In this study, we excluded patients with a clinical diagnosis of CHF at baseline, so our findings cannot be applied to those patients with CKD and underlying CHF.

In a recent paper we have found that a low SBP (less than 120 mmHg) was associated with worse survival in patients with CKD, nonetheless that patient population had more cardiovascular comorbidities at baseline (especially CHF). Based on our current findings we think studies evaluating the optimal BP target in older patients with CKD might need to differentiate among patients with and without heart failure and LVH [25].

Although this was an observational study, the follow up was extensive, given the unique geographical characteristics of this population. Most patients receive their care in one of two local clinics and are admitted to one or two hospitals which share electronic medical records. This allows for close follow up of outcomes of interest.

Among the limitations of this study we cite the retrospective design, small number of patients, lack of significant number of minorities, inability to assess for reversibility of LVH changes, use of ECG and echocardiographic criteria together, although in CKD patients LVH on electrocardiogram is associated with a higher increase in CV mortality than other traditional risk factors [26].

## Conclusion

The combination of higher systolic blood pressure and LVH might identify older patients with CKD at higher risk of cardiovascular outcomes. These results should be confirmed with larger studies.
